# Recent Advances in Photoinduced Electron Transfer Processes of Fullerene-Based Molecular Assemblies and Nanocomposites

**DOI:** 10.3390/molecules17055816

**Published:** 2012-05-16

**Authors:** Osamu Ito, Francis D’Souza

**Affiliations:** 1 CarbonPhotoScience, Kita-Nakayama, 2-1-6, Izumi-ku, Sendai 981-3215, Japan; 2 Department of Chemistry, University of North Texas, 1155 Union Circle, #305070, Denton, TX 76203, USA

**Keywords:** fullerenes, porphyrins, phthalocyanine, single-wall carbon nanotubes, electron-transfer, photoelectrochemistry

## Abstract

Photosensitized electron-transfer processes of fullerenes hybridized with electron donating or other electron accepting molecules have been surveyed in this review on the basis of the recent results reported mainly from our laboratories. Fullerenes act as photo-sensitizing electron acceptors with respect to a wide variety of electron donors; in addition, fullerenes in the ground state also act as good electron acceptors in the presence of light-absorbing electron donors such as porphyrins. With single-wall carbon nanotubes (SWCNTs), the photoexcited fullerenes act as electron acceptor. In the case of triple fullerene/porphyrin/SWCNT architectures, the photoexcited porphyrins act as electron donors toward the fullerene and SWCNT. These mechanisms are rationalized with the molecular orbital considerations performed for these huge supramolecules. For the confirmation of the electron transfer processes, transient absorption methods have been used, in addition to time-resolved fluorescence spectral measurements. The kinetic data obtained in solution are found to be quite useful to predict the efficiencies of photovoltaic cells.

## 1. Introduction

**Figure 1:**
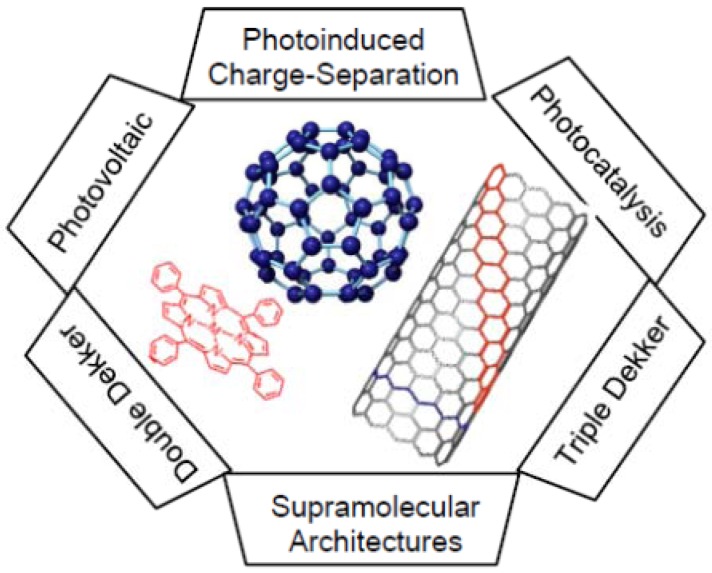
Functionalized fullerenes with porphyrins and SWCNTs, which induce charge separation by light illumination applicable for photo-voltaic and photosensing systems.

The study of photoinduced electron transfer in donor-acceptor systems is one of the growing research areas driven primarily by solar energy conversion [1], ultimately used in the construction of molecular electronic and optoelectronic devices [2,3]. Among the donor-acceptor systems, porphyrin-fullerene systems are one of the most widely studied classes of compounds due to their rich photo- and redox chemical properties [4,5,6,7,8,9,10,11,12,13,14,15,16,17,18,19,20,21,22,23,24,25]. Both covalently linked systems and non-covalent systems assembled via metal-ligand coordination or π−π stacking have been elegantly designed and studied. Fullerenes, owing to their spherical shape, possess high electron-affinity and require small reorganization energy in the electron-transfer processes [21]. Consequently, in donor-acceptor systems, fullerenes tend to accelerate forward electron transfer and slow down backward electron transfer, resulting in the formation of long-lived charge-separated (CS) states [9,19,20]. This is a key factor for utilizing fullerenes in building the solar energy conversion devices [21,22,23]. The electron-rich macrocyclic compounds such as porphyrins have been widely used as biomimetic photosensitizing electron donor in these studies, since they absorb lights over wide wavelengths in the visible region and exhibit favorable redox potentials [3,4]. In the present review, we include chemically functionalized single-wall carbon nanotubes (SWCNTs) as photoactive electron-conductive materials [26,27] for the development of photovolatic cells. We expect that a combination of these three kinds of molecules, acting as light-harvesting donors-acceptor systems, will be useful materials for photocatalytic and light-energy conversion applications, as shown in Figure 1.

## 2. Fullerene-Porphyrin Systems

We describe here the recent developments in the construction of self-assembled supramolecular donor-acceptor conjugates with porphyrin as donor and fullerene (C_60_) as electron acceptor by adopting different self-assembly mechanisms. The typical examples are the functionalized fullerenes coordinated to the central metal atom of the porphyrins [28]. In addition, the photoinduced charge-separation of the chemically functionalized fullerenes with porphyrin via the covalent bond can be controlled by the additive effect involving coordination to the central metal of the porphyrin [29,30,31,32,33]. 

### 2.1. Fullerene-Porphyrin Coordination Systems

**Figure 2:**
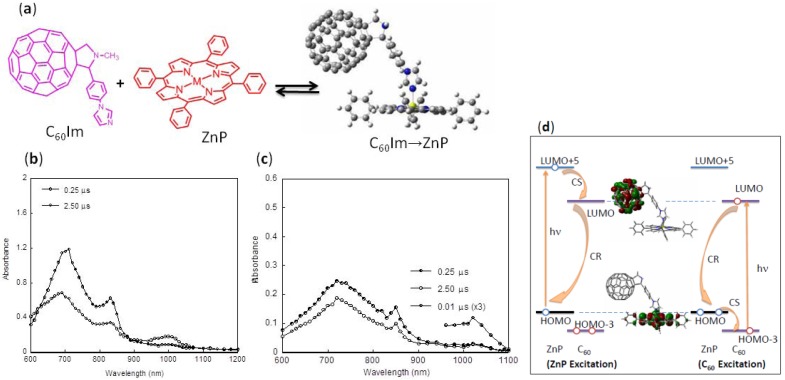
(**a**) Equilibrium between C_60_Im and ZnP [for C_60_Im→ZnP, Zn (yellow), N (blue), C (grey), H (white)]; (**b** and **c**) Nanosecond transient absorption spectra and time profiles in Ar-saturated solution; (**b**) in PhC≡N and (**c**) in *o*-DCB (adopted from [36]); (**d**) Energy diagram and HOMO and LUMO of C_60_Im→ZnP complex (CS; charge separation and CR; charge recombination) constructed from the data in [36].

Usually in polar solvents, a mixture of pristine C_60_ and zinc or magnesium porphyrins (ZnP or MgP) shows intermolecular electron transfer by visible light illumination, giving their radical anion (C_60_^∙−^) and radical cation (MP^∙+^), respectively, as revealed directly by the transient absorption spectral measurements [34,35]. This is also recognized when C_60_ is functionalized with imidazole (C_60_Im) or pyridine (C_60_Py) entities; that is, they undergo intermolecular electron transfer as revealed by the slow rises of the C_60_^∙−^Im at 1,000 nm and MP^∙+^ at 620 nm as shown in the transient absorption spectra and their time-profiles in polar solvents such as PhC≡N (Figure 2b) [36,37,38]. Under the usual concentrations of C_60_Im and MP, the electron transfer takes place via their triplet excited states (^3^C_60_*Im at 700 nm and ^3^MP* at 820 nm), which also show a slow decay, confirming the intermolecular electron transfer. These findings are evidence for the destruction of the coordination complex via blocking of the metal atom of MP by the solvent PhC≡N molecules. From the time profiles for the decay of ^3^C_60_*Im and the rise of C_60_^∙−^Im (inset of Figure 2b), the pseudo-first order rate constant can be evaluated to be *ca*. 3 × 10^5^ s^−1^, giving the second-order rate constant of 3 × 10^9^ M^−1^·s^−1^ for concentrations of 0.1 mM.

When both C_60_ and ZnP are in high concentrations (>100 mM), it is notable that the intermolecular electron transfer takes place via the singlet excited states (^1^C_60_* and ^1^ZnP*), as evidenced by their fluorescence quenching and the quick rise of C_60_^∙−^ and ZnP^∙+^; furthermore, these C_60_^∙−^ and ZnP^∙+^ decay quickly within 10 ns, even in PhC≡N, due to the radical ion-pairing (RIP) with the singlet-spin character, in which the back electron transfer usually occurs very fast [39]. 

In *o*-dichlorobenzene (*o*-DCB), however, the supramolecular complex (C_60_Im→MP) exists as a major equilibrium species in solution (*K*_complex_ = 11 × 10^4^ M^−1^); thus, quite different features are found in the transient absorption spectra, as shown in Figure 2c. The time-profile of the C_60_^∙−^Im peak at 1,020 nm reveals a sharp rise-and-decay curve within 100 ns immediately after the ns laser-light pulse, although the absorption peak of the ZnP^∙+^ in the 600–680 nm region is hidden by the huge absorptions of the C_60_*Im at 700 nm. The sharp rise of the C_60_^∙−^Im corresponds to the ZnP-fluorescence decay within 0.2 ns (*k*_CS_ = 1.7 × 10^10^ s^−1^), suggesting the charge-separation (CS) process takes place via ^1^ZnP* entity within the coordination complex. From the decay of the C_60_^∙−^Im within ca. 20 ns, the rate of charge recombination, *k*_CR_, in the studied dyads was obtained to be *k*_CR_ = ca. 5 × 10^7^ s^−1^, which is also reasonable due to the close distance between the electron and hole in the C_60_^∙−^Im→ZnP^∙+^. 

In the picosecond time region, the charge-separation in such coordination complexes takes place very rapidly, within 200 ps, with the rise of the radical ions with concomitant decay of the S_n_→S_1_ transition. Then the radical ions decay with ca. 5 × 10^7^ s^−1^, giving the lifetime of the radical ion pair (*τ*_RIP_) to be 20 ns in non-polar solvents. 

Figure 2d shows the molecular-orbital (MO) energy-diagram for photoinduced CS and CR processes of the C_60_Im→ZnP complex via ^1^ZnP* entity with their HOMO and LUMO; the HOMO is mainly localized on the ZnP moiety, whereas the LUMO is localized on the C_60_ moiety, suggesting the most stable CS state is the RIP like as C_60_^∙−^Im→ZnP^∙+^. When the ZnP is photo-excited, the HOMO-electron of the ZnP is risen to the upper LUMO+5 localizing on the ZnP, from which the risen electron transferred to the LUMO localizing on the C_60_ moiety, leaving the hole on the ZnP-HOMO, which gives C_60_^∙−^Im→ZnP^∙+^ as a stable RIP. When the C_60_ moiety is photo-excited, the electron of the HOMO-3 on the C_60_ moiety is risen to the LUMO of C_60_, subsequently, the half-vacant HOMO-3 abstracts an electron from the HOMO level of the ZnP moiety, giving the C_60_^∙−^Im→ZnP^∙+^. 

From the redox potentials, the free energy change for photoinduced intermolecular electron transfer for the mixture system (−Δ*G*_PET_) can be calculated to be 0.3 eV via either the ^3^C_60_*Im (ca. 1.5 eV) or ^3^ZnP*(ca. 1.5 eV), whereas the free energy for charge recombination (−Δ*G*_CR_) is calculated to be 1.2 eV, which also represents the energy of the RIP. Since the reorganization energy for electron transfer of the spherical C_60_ molecule is reported to be as small as 0.5 eV, the CS process positions almost in the top region of the Marcus bell curve, whereas the CR process positions in the deep inverted region. 

From the redox potentials, the free energy change for intramolecular charge separation (Δ*G*_CS_) for the C_60_Im→ZnP complex can be calculated to be 0.5 eV via the ^1^C_60_*Im (ca. 1.8 eV) and to be 0.8 eV via ^1^ZnP* (ca. 2.1 eV). Thus, the CS process positions almost at the top region of the Marcus bell curve, whereas the CR process is still in the deep inverted region. Therefore, the lifetime of the C_60_^∙−^Im→ZnP^∙+^ is prolonged over 1 ns.

From the energies of the excited states, the energy transfer from ^1^ZnP* to C_60_Im is also possible; although the C_60_-fluorescence is not observed after the decay of the ZnP-fluorescence, the absorption bands of ^3^C_60_*Im is observed as shown in Figure 2c, suggesting that the energy transfer might occur concurrently with the CS within C_60_Im→ZnP.

### 2.2. Fullerene-Porphyrin Charge-Transfer Systems

**Figure 3:**
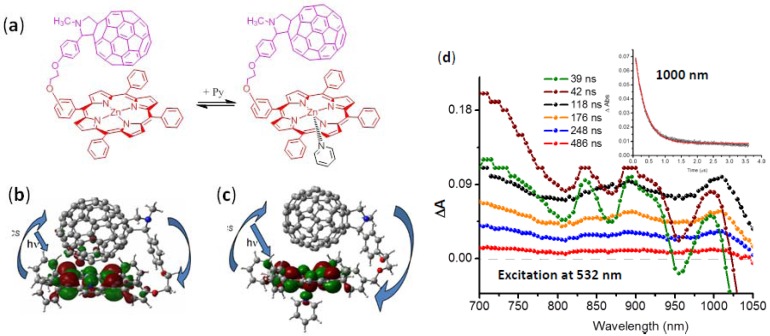
Effect of pyridine (Py) addition on the C_60_~ZnP dyad; (**a**) equilibrium; (**b**) HOMO of C_60_~ ZnP; (**c**) HOMO of C_60_~ ZnP coordinated with Py; (**d**) noanosecond transient absorption spectra of C_60_~ZnP in the presence of excess Py in Ar-saturated *o*-DCB. Inset: Time profile at 1,000 nm (adopted from [30] and [40]).

In another study, the pyridine coordination effect on the charge separation and charge recombination of covalently linked C_60_~ZnP dyads is investigated (Figure 3a) [30]. In o-DCB, photoexcitation gives the C_60_^∙−^~ZnP^∙+^ via C_60_~^1^ZnP* and/or ^1^C_60_*~ZnP in ca. 5 × 10^9^ s^−1^ as revealed by the fluorescence lifetime and ps-transient absorption spectra. The C_60_^∙−^~ZnP^∙+^ displays relatively short lifetimes similar to the coordination complex (ca. 20 ns in Figure 2c), because of the vicinity of C_60_^∙−^ and ZnP^∙+^. However, on addition of a small amount of pyridine to coordinate the Zn ion of the ZnP moiety in o-DCB solution, the transient absorption spectra changes to Figure 3d [40]. After 10 ns laser-light pulse, the 1,000 nm peak reaches a maximum at 100 ns. Before 100 ns, sharp peaks appear at 700 nm and 800–900 nm, which can be attributed to ^3^C_60_* and ^3^ZnP*, respectively. A sharp peak is also observed at 1,000 nm, which can be ascribed to C_60_^∙−^. After 100 ns, the broad bands are found at 1,000 nm and 880 nm, which are also attributed to C_60_^∙−^ and ZnP^∙+^, respectively, although the solvation of the RIP (C_60_^∙−^ and ZnP^∙+^) may change from that before 100 ns. The τ_RIP_ of the C_60_^∙−^ and ZnP^∙+^ prolongs up to 200 ns (Figure 3d). 

The MO calculations of the C_60_~ZnP are shown in Figure 3b, in which the optimized structure indicates the short distance between the C_60_ sphere and ZnP plane in C_60_~ZnP. Earlier, such calculations on fullerene-based supramolecular nano-architectures predicted geometries and the frontier molecular orbitals to a certain accuracy at the B3LYP/3-21G(*) level [41]. In the HOMO, although the electron mainly localizes on the ZnP moiety, a small amount of the electron is distributed to the near-side of the C_60_ sphere, suggesting charge-transfer (CT) interactions exist in the ground state. Therefore, the observed transient species must be short-lived, because of the excited state of the CT state, which is considered to be delocalized RIP in the whole C_60_~ZnP , but not pure RIP like as the HOMO-LUMO in Figure 2c. 

On the other hand, the coordination of the pyridine at the ZnP of the opposite side to the C_60_ sphere tends to elongate the distance between the C_60_ sphere and the ZnP plane as revealed in the optimized structure by ~0.5 Å as shown in Figure 3c; furthermore, such coordination makes the HOMO predominantly localized on the ZnP moiety (Figure 3c), destroying the CT interaction. Instead, the electron distributes to the coordinated pyridine, which improves the hole-delocalization in the RIP. These characters of the HOMO result in the prolonging the RIP, as observed in Figure 3d. 

The coordination of the additional pyridine to the ZnP plane on the same side to the C_60_ sphere can separate the distance between the both entities (right structure in Figure 3a); then, the photo-excitation produces pure RIP (C_60_**^∙^^−^**~ZnP**^∙+^**) and its CR rate is slowed down, which may be one of the reasons for long-lived C_60_**^∙^^−^**~ZnP**^∙+^** in Figure 3d. 

In addition, other electronic factors can be considered to be responsible for this observation, because of the broadening of the transient bands after 100 ns suggests the characteristic interactions between the pyridine molecules and C_60_ molecule, and sometimes induces aggregation of C_60_ molecules resulting in fine particles. 

When the C_60_ moiety with a pyridine unit in C_60_~ZnP is employed, intramolecular coordination of the Py to ZnP forms a parachute-like structure between C_60_ and ZnP in *o*-DCB; in such a parachute-like structure, strong interaction between two entities would be anticipated (Figure 2a). Indeed, similar transient spectra to Figure 2c are observed. On addition of excess pyridine molecules, intermolecular coordination bond is destroyed, giving loose structures similar to Figure 3c, and transient spectra similar to Figure 3d with long-living RIP [29]. 

Summarizing this section, for the photoinduced electron transfer processes for the intermolecular, supramolecular, and flexible covalent bonding systems between C_60_ and porphyrins, the appearance of the transient absorption band of C_60_^∙−^ at 1,000 nm is diagnostic evidence of the intermolecular electron transfer and intramolecular charge-separation. These mechanisms and the lifetimes of the C_60_^∙−^ entities in these systems, which are sensitively changed by the delicate molecular structures and environments guided by the MO calculations, are important findings to utilize in the construction the photo-active energy harvesting or fuel production devices.

## 3. Fullerene-SWCNT Systems

Single walled carbon nanotubes (SWCNTs) are emerging new materials for diverse applications including electronics, composites, and biosensors [26,27]. The unique structural properties have made them novel materials in nanotechnology. On combination with light-sensitive molecules, SWCNTs can be modified as light-energy harvesting materials over a wide wavelength range [9,21,23]. Accordingly, various light-sensitive devices and photovoltaic applications using chemically modified SWCNTs have been reported [3,4,5,6,7,8,9,10,11,12,13]. When SWCNT samples including both metallic and semiconducting tubes with different chirality indices are employed as “a material”, no meaningful structure-reactivity relationship could be derived. However, we recently overcame this hurdle by employing single-diameter enriched SWCNT(*n*,*m*), that allowed us to consider each SWCNT as “a molecule”, which may open a new paradigm for chemically modified SWCNT systems [6]. Thus, by constructing self-assembled supramolecular C_60_/SWCNT(*n*,*m*) hybrids and C_60_→ZnP/SWCNT(*n*,*m*), we investigated the photoinduced charge separation processes in solution by various photochemical and photophysical techniques. We also constructed photovoltaic cells by adsorbing the donor-acceptor nanohybrids on an appropriate electrode surface decorated with semiconductor nanoparticles.

### 3.1. Fullerene-SWCNT Systems

A quick literature survey shows that only a few examples of studies involving SWCNTs with a combination of C_60_ for probing photoinduced electron transfer have been reported [42,43,44,45,46]. Moreover, no systematic study has ever been done on enriched semi-conducting SWCNT(*n*,*m*) for their ability to be electron donors or acceptors. 

**Figure 4:**
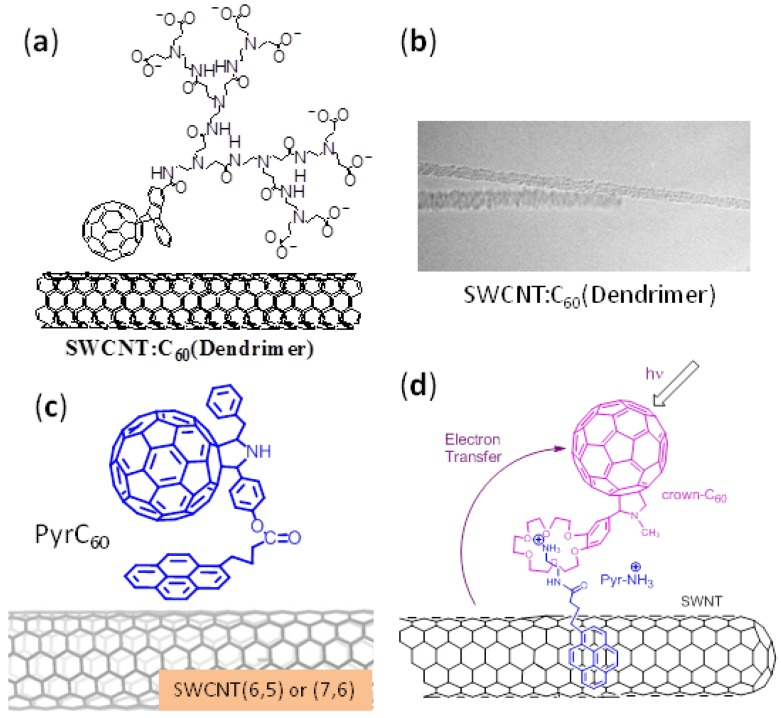
(**a**) SWCNT/C_60_-dendrimers; (**b**)TEM image; (**c**) C_60_Pyr /SWCNT assembled via π−π interaction (SWCNT(6,5) and SWCNT(7,6)); and (**d**) SWCNT/pyrNH_3_^+^/Crown-C_60_ assembled via cation-dipole inclusion complex formation (adopted from [43,44,45,46]).

To accomplish the non-covalent supramolecular attachment, C_60_-dendrimers are mixed with SWCNT in solution [43,44]. Figure 4a is an example of a water-soluble C_60_-dendrimer, which tends to surround SWCNT with π–π interactions and also with alkyl chain entanglements. Indeed, the TEM images in Figure 4b show the rough surface along SWCNT, suggesting the adsorption of the C_60_-dendrimers [32]. 

We also newly functionalized C_60_ to possess a pyrene moiety (C_60_Pyr) as shown in Figure 4c. To produce C_60_Pyr/SWCNT(*n,m*) nanohybrids, C_60_Pyr were mixed with SWCNT(*n*,*m*) in DMF [46]. After purification by repeated sonication and centrifugation, un-complexed C_60_Pyr were eliminated, increasing the content of attached C_60_Pyr molecules [46]. In C_60_Pyr/SWCNT nanohybrids thus produced, the Pyr moiety attaches to the SWCNT surface via π−π interactions (Figure 4c). In addition, the π orbitals of the C_60_ sphere may also assist the complex formation via the π−π interaction. 

As a third approach, a pyrene functionalized with an alkylammonium cation, PyrNH_3_^+^, is stacked to SWCNT via π−π interaction, forming SWCNT/PyrNH_3_^+^, to which a benzo-18-crown-6 functionalized C_60_ (Crown-C_60_) is self-assembled via cation-dipole interactions, finally generating SWCNT/PyrNH_3_^+^/Crown-C_60_ as shown in Figure 4d [45]. The SWCNT/C_60_ nanohybrids generated by these approaches are characterized by TEM and thermal analysis methods. 

### 3.2. Steady-State Spectral Studies of Fullerene-SWCNT Systems

**Figure 5:**
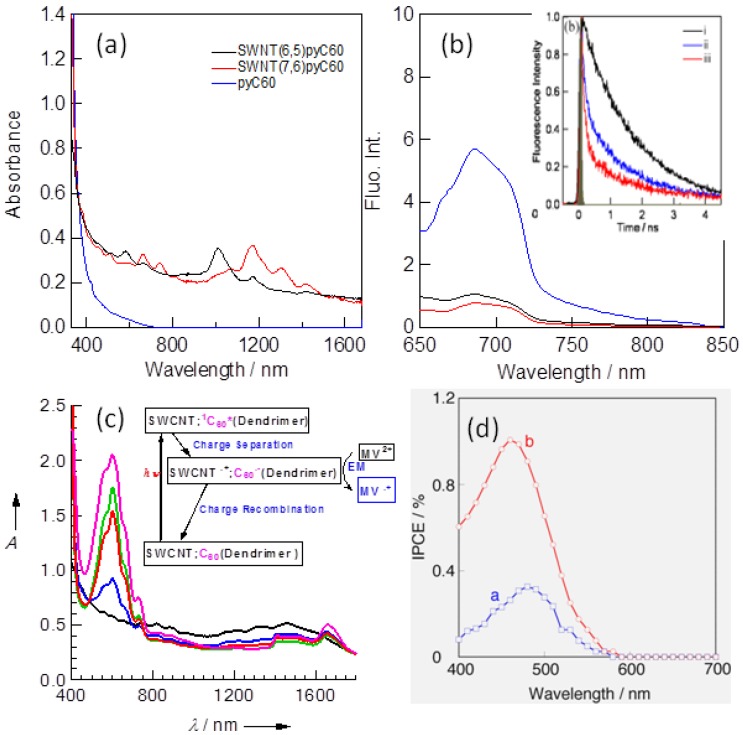
(**a**) Steady-state absorption spectra; blue for of C_60_Pyr/SWCNT(6,5) and red for C_60_Pyr/SWCNT(7,6), black for black forPyrC_60_; (**b**) fluorescence spectra and time profiles; (**c**)methyl viologen radical cation (MV^•+^) accumulation by the light excitation of C_60_ in SWCNT/C_60_-dendrimer (MV^2+^ and BNAH) in Ar-saturated H_2_O; (**d**) IPCE for voltaic cell of FTO/SnO_2_/C_60_Pyr/SWCNT(*n*,*m*) in the presence of LiI/I_2_ in acetonitrile solution (CH_3_C≡N) (adopted from [46]).

Figure 5a shows the steady-state absorption spectra of C_60_Pyr/SWCNT(*n*,*m*) in the wide region from UV-Visible to NIR; the blue spectrum (ii) for C_60_Pyr/SWCNT(6,5) shows the absorption peak at ca. 1,000 nm in addition to the absorption of C_60_Pyr [black spectrum (i)] in the UV and Visible region, whereas the red spectrum (iii) for C_60_Pyr/SWCNT(7,6) shows a peak at 1,200 nm. These longest absorption peaks correspond to the band gaps of the semiconducting SWCNT; the larger diameter one has narrower band gap than that of the smaller one. The 500–400 nm band is attributed to the characteristic absorption of the C_60_ moiety.

Figure 5b shows the steady-state fluorescence spectra and time profiles of the C_60_ moiety in C_60_Pyr/SWCNT(*n*,*m*) hybrids. Compared with the fluorescence intensity of free C_60_Pyr at 680 nm, the intensities decrease down to 1/5, suggesting that photo-physical events originate from the ^1^C_60_*Pyr/SWCNT state. The fluorescence time profiles gave more reliable information than the steady-state fluorescence measurements, because the fluorescence time profiles do not depend on the concentration and light scattering. Quick decays of the C_60_ fluorescence give the short lifetimes of ca. 200 ps, which correspond to the fluorescence quenching rate constant of ca. 5 × 10^9^ s^−1^. 

In order to estimate the photoinduced CS events, it is most convenient to observe the electron-pooling in solution on addition of methyl viologen (MV^2+^) in the presence of appropriate hole-transfer reagents such as 1-benzyl-1,4-dihydronicotinamide (BNAH). On selective photo-excitation of the C_60_ moiety in the SWCNT/C_60_-dendrimers in the presence of MV^2+^ and BNAH, the MV^∙+^ absorption band intensity at 620 nm is accumulated and preserved as shown in Figure 5c. This implies that MV^2+^ accepts an electron from the C_60_^∙−^, which can be formed via the CS process between the ^1^C_60_* and SWCNT under photo-excitation of the C_60_ moiety as shown in scheme in inset of Figure 5c. This suggests that the photocatalytic reduction of MV^2+^ takes place by the photo-excitation of C_60_Pyr/SWCNT(*n*,*m*) with consuming BNAH. 

A photovoltaic cell is also constructed by attaching C_60_Pyr/SWCNT(*n*,*m*) to the SnO_2_/FTO surface and the incident photon to current conversion efficiency (IPCE%) spectra are measured. As shown in Figure 5d, an IPCE peak is observed at 460 nm, which is in agreement with the absorption peak of the C_60_ moiety, supporting the electron transfer starts from the light absorption of the C_60_ moiety, but not from SWCNT, although the NIR region data is lacking. Although the IPCE values are not high, a relatively higher value is obtained for C_60_Pyr/SWCNT(7,6) compared to C_60_Pyr/SWCNT(6,5), which can be interpreted with the wider band-gap and higher conductivity of SWCNT(7,6) [46].

### 3.3. Direct Evidence for Charge Separation of Fullerene-SWCNT Systems

**Figure 6:**
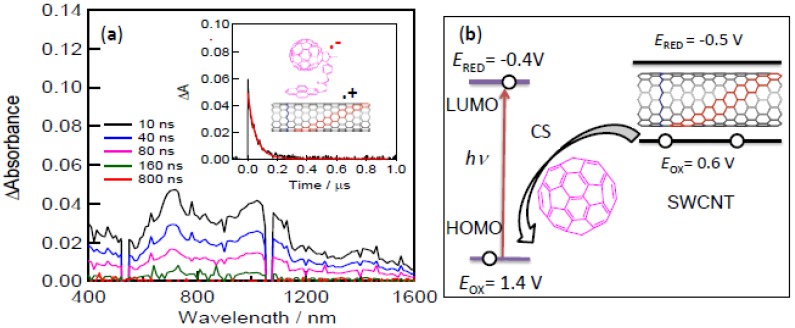
(**a**) Nanosecond transient absorption spectra of SWCNT(7,6)/PyrC_60_ in Ar-saturated *o*-DCB (negative spikes at 532 and 1024 nm are due to the scattering of YAG laser light). Inset: Time profile at 1,000 nm; (**b**) Energy diagram for charge separation of SWCNT(7,6)/PyrC_60_ induced by the photo-excitation of the C_60_ moiety (adopted from [46]).

Nanosecond transient absorption spectra of C_60_Pyr/SWCNT(7,6) are measured by the selective photo-excitation of the C_60_ moiety. Figure 6a shows the observed spectra for the C_60_Pyr/SWCNT(7,6) hybrid; similar spectra are obtained for the C_60_Pyr/SWCNT(6,5) hybrid. The absorption bands around 1,000 nm corresponding to C_60_^∙−^Pyr are clearly recognized with weak peaks in the 1,200–1,600 nm region possibly due to the one-electron oxidized SWCNTs, suggesting the formation of C_60_^∙−^Pyr/SWCNT(7,6)^∙+^. Appearance of the 740-nm band corresponding to ^3^C_60_* indicates the loosely bound or free C_60_Pyr. The CR rate (*k*_CR_) evaluated from the C_60_^∙−^Pyr decay (inset of Figure 6a) is 1.80 × 10^7^ s^−1^ for C_60_^∙−^Pyr/SWCNT(7,6)^∙+^; similarly, the *k*_CR_ value evaluated to be 1.21 × 10^7^ s^−1^ for C_60_^∙−^Pyr/SWCNT(6,5)^∙+^. The larger *k*_CR_ value for C_60_^∙−^/SWCNT(6,5)^∙+^ may be related to the higher electron-conductivity of SWCNT(7,6). The ratios of *k*_CS_/*k*_CR_, which are measure of the exploitable electron-hole of the CS state, are found to be ~360, suggesting feasibility of these nanohybrids for the construction of devices using the CS states. 

To further understand the excited state events originating from the C_60_ moiety, an energy level diagram is constructed from the reported electrochemical redox data [47]. As shown in Figure 6b, since the LUMO of C_60_ is lower than the conduction band of SWCNT, a photo-excited electron of C_60_ cannot transfer to the conduction band SWCNT, whereas an electron of the valence band of SWCNT(*n,m*) can be poured down to the half-vacant C_60_-HOMO, generating the charge-separated RIP state, C_60_^∙−^Pyr/SWCNT(*m*,*n*)^∙+^. The energies of C_60_^∙−^Pyr/SWCNT(*m*,*n*)^∙+^ can be evaluated to be 0.9–1.0 eV from the difference between *E*_OX(SWCNT)_ and *E*_RED(C60)_. Thus, the free-energy changes for CS process (*ΔG*_CS_) via ^1^C_60_*Pyr (1.80 eV) are negative (*ΔG*_CS_ = −0.8 – −0.9 eV), predicting exothermic CS process. 

Usually, the fluorescence quenching observed in Figure 5b suggests both the energy transfer and electron transfer. However, direct energy transfer from ^1^C_60_* (half-occupied LUMO) to the conduction-band of SWCNT(*n,m*) is not efficient, via electron-exchange mechanism since an endothermic process is anticipated since E_LUMO(C60)_ < E_conduction-band(SWCNT)_ in Figure 6b. Resonance energy transfer from ^1^C_60_* to SWCNT(*n*,*m*) may also not be efficient, because the C_60_-fluorescence band (650–730 nm) is far from the SWCNT(*n*,*m*)-absorption bands (1,000–1,200 nm); furthermore, the spectroscopic overlap is not large, because the C_60_-fluorescence intensity and absorption intensity of the semiconducting SWCNTs in the Visible region are both very low. Therefore, the fluorescence quenching of the C_60_ moiety can be attributed to the CS process via ^1^C_60_* as confirmed by the transient absorption spectra.

From the energy level diagram (Figure 6b), an electron-transfer from the conduction band of SWCNT(*n*,*m*) to the LUMO of C_60_ is plausible after the excitation of SWCNT(*n*,*m*). However, the lifetimes of the excited state of the SWCNT(*n*,*m*) are very short (<1 ps), relaxing to their ground states before an electron transfer to the vicinal C_60_.

To summarize this section, stable nanohybrids are produced with functionalized fullerenes with SWCNT(*n*,*m*), positioning the C_60_ spheres in the close proximity to the surface of SWCNT, making the two-layer nanohybrids. Such nanohybrids are found to be stable by the TEM images and steady-state optical spectral studies. Photoinduced CS processes in the supramolecular C_60_/SWCNT(*n*,*m*) hybrids having (6,5)- and (7,6)-enriched SWCNTs was demonstrated with the help of time-resolved fluorescence studies. The nanosecond transient absorption technique confirms the CS products, C_60_^∙−^/SWCNT^∙+^, which upon surface modification operated as photovoltaic cell under solar visible light irradiation.

## 4. Fullerene-Porphyrin-SWCNT Systems

Since the absorption intensity of the C_60_ moiety in the Visible region is not high enough to utilize them in solar light harvesting, we tried to introduce the photo-sensitizers such as porphyrins to the C_60_-SWCNT(*n*,*m*) systems [48]. Three-layer supramolecular hybrids, C_60_-porphyrin-SWCNT are constructed from semiconducting (7,6)- and (6,5)-enriched SWCNTs. 

### 4.1. Molecular Structure and Energy Level Diagram

**Figure 7:**
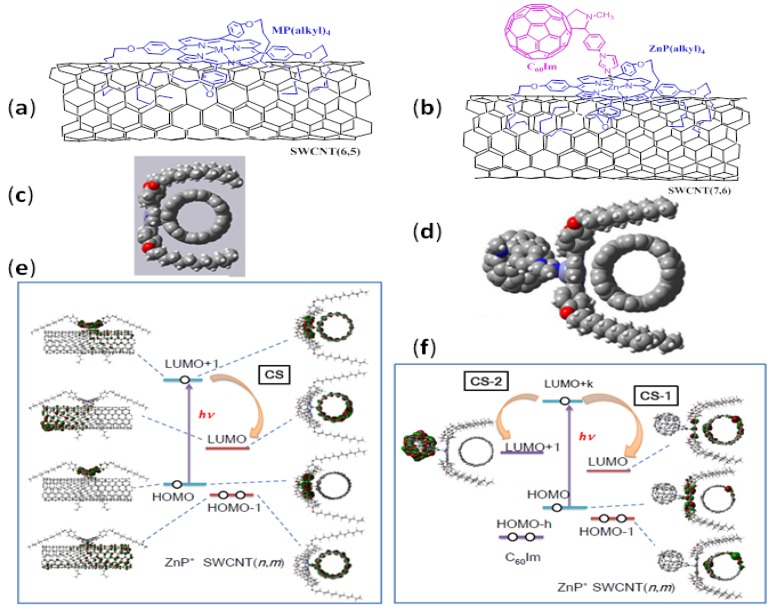
Illustration of (**a**) ZnP(alkyl)_4_/SWCNT(*n*,*m*) and (**b**) C_60_Im→ZnP(alkyl)_4_/ SWCNT(*n*,*m*) donor-acceptor nanohybrids. Edge-on view of the optimized structures at the B3LYP/3-21G(*) level of (**c**) ZnP(alkyl)_4_ interacting with SWCNT(7,6); and (**d**) C_60_Im→ZnP(alkyl)_4_)/SWCNT; C (gray), H (white), O (red), Zn (light blue), N (blue). For the calculation, 300 carbon atoms are used to build SWCNT(7,6). Energy diagrams and electron distributions for (**e**) ZnP(alkyl)_4_)/SWCNT(7,6) and (**f**) C_60_Im→ZnP(alkyl)_4_)/ SWCNT(7,6) calculated at the B3LYP/3-21G(*) level (adopted from [48]).

C_60_-porphyrin-SWCNT are constructed via a supramolecular method; first, long alkyl-chain substituted zinc porphyrins [tetrakis(4-dodecylphenyl) zinc porphyrins (ZnP(alkyl)_4_] are mixed with SWCNT(*n,m*) in organic solvents. The ZnP(alkyl)_4_/SWCNT(*n,m*) hybrids are formed via the intermolecular alkyl-π and π−π interactions between the ZnP(alkyl)_4_ and SWCNT(*n,m*) (Figure 7a). The formation of the ZnP(alkyl)_4_/SWCNT(*n,m*) hybrids are confirmed by the ZnP-fluorescence quenching in organic solution, suggesting occurrence of some photophysical events via ^1^ZnP*. On addition of C_60_Im to ZnP(alkyl)_4_/SWCNT(*n,m*), the C_60_Im→ZnP(alkyl)_4_/SWCNT(*n*,*m*) is formed via the coordination of C_60_Im to Zn ion (Figure 7b).

The intermolecular alkyl-π and π−π interactions and relative orientation of (ZnP(alkyl)_4_) on the surface of the semiconducting SWCNT can be visualized by performing DFT-MO calculations [41]. The edge-on view in Figure 7c shows an optimized structure of ZnP(alkyl)_4_/SWCNT(7,6), which clearly reveals that the ZnP π-plane is interacting with the surface of SWCNT(7,6); the distance between Zn and nearest carbon of the nanotube is found to be 2.1 Å, suggesting close association of the entities [48]. In addition, the alkyl chains also get their arms around the SWCNT(7,6). 

The DFT-MO calculations of C_60_Im→ZnP(alkyl)_4_/SWCNT(7,6) show an optimized structure including C_60_Im-Zn axial coordination in addition to ZnP(alkyl)_4_/SWCNT(7,6) as shown in Figure 7d. The optimized distance Im→Zn ≈ 2.0 Å is similar to that reported earlier for C_60_Im→ZnP dyad computationally [41] and by X-ray structure [49]. On the other hand, the Zn-SWCNT distance increased to about 2.2 Å due to slight pulling of the Zn from the porphyrin macrocycle due to axial coordination.

The frontier orbitals for the two-layer ZnP(alkyl)_4_/SWCNT(7,6) system are also shown in Figure 7e. The majority of the HOMO is found on the ZnP entity, while the majority of the HOMO-1 is found on SWCNT(7,6). The LUMO was found on SWCNT(7,6), whereas the LUMO+1 is mainly located on the ZnP entity and partially on SWCNT(7,6). The pair of the HOMO and the LUMO corresponds to the most stable CS state as described to be ZnP^∙+^(alkyl)_4_/SWCNT^∙−^, whereas the pair of the HOMO and the LUMO+1 corresponds to the local excitation of the ZnP moiety. Consequently, excitation of the HOMO on the ZnP moiety rises an electron to the LUMO+1 within ZnP, from which the electron falls down to the LUMO of SWCNT(7,6), leaving a hole on the HOMO of ZnP, which results in ZnP^∙+^(alkyl)_4_/SWCNT^∙−^ via ^1^ZnP*. If the excited state of SWCNT(7,6) is long enough, electron on the ZnP falls down to the vacant valence bond of SWCNT(7,6), giving ZnP^∙+^(alkyl)_4_/SWCNT^∙−^ via ^1^SWCNT*, which will open the utilization of the NIR light for solar energy conversion. 

The frontier orbitals for the three-layered C_60_Im→ZnP(alkyl)_4_/SWCNT(7,6) system are also shown in Figure 7f. The majority of the HOMO was found to be on ZnP entity, while the majority of the HOMO-1 was found to be on SWCNT(7,6). The LUMO was found to be mainly localized on SWCNT(7,6) and partially on ZnP, whereas the LUMO+1 is fully located on the C_60_ entity. Pair of the HOMO and the LUMO corresponds to the CS state as described around ZnP^∙+^(alkyl)_4_/ SWCNT^∙−^(7,6), while having slight CT character. On the other hand, pair of the HOMO and the LUMO+1 corresponds to the CS state as C_60_^∙−^Im→ZnP^∙+^(alkyl)_4_. From the calculated MO energy levels, ZnP^∙+^(alkyl)_4_/ SWCNT^∙−^ is more stable than C_60_^∙−^Im→ZnP^∙+^(alkyl)_4_. When the electron on the LUMO+k (k>5) of the ZnP moiety transfers to the LUMO of SWCNT, ZnP^∙+^(alkyl)_4_/SWCNT^∙−^ can be produced, which is denoted as CS-1 process in Figure 7f. On the other hand, when the electron on the LUMO+k on the ZnP moiety transfers to LUMO+1 of C_60_, C_60_^∙−^Im→ZnP^∙+^(alkyl)_4_ can be produced as CS-2 process. The photo-excitation of C_60_ makes a hole on the HOMO-h (h > 10), to which the HOMO-electron of ZnP flows down to the hole on the HOMO-h of C_60_, giving C_60_^∙−^Im→ZnP^∙+^(alkyl)_4_, too. If the lifetime of the excited state of SWCNT(7,6) is long enough, electron on the ZnP falls down to the vacant valence bond of SWCNT(7,6), giving ZnP^∙+^(alkyl)_4_/SWCNT^∙−^ via ^1^SWCNT*. 

The energy diagrams can be constructed from the reported redox potentials for C_60_Im, MP(alkyl)_4_, SWCNT(6,5) and SWCNT(7,6) in DMF; they are almost the same as the HOMO and LUMO in Figure 7e and Figure 7f, excepting the order of the HOMO of ZnP and the HOMO-1 of SWCNT, which makes electron trasfer from the SWCNT to half-vacant ZnP, giving ZnP^∙−^(alkyl)_4_/SWCNT^∙+^. 

Experimentally, these nanohybrids are characterized by TEM imaging, steady-state absorption and fluorescence spectra. The ZnP-fluorescence intensity is quenched on complexing with SWCNT(*n*,*m*) accompanying the shortening of the fluorescence lifetimes (τ_F_) of the ^1^ZnP*(alkyl)_4_ in two-layered nanohybrids (ZnP(alkyl)_4_/SWCNT), giving the k_CS_ values to produce C_60_^∙−^Im→ZnP^∙+^(alkyl)_4_ in the range of (5–6) × 10^9^ s^−1^. On further addition of C_60_Im, the ZnP-fluorescence intensity is additionally quenched. The time-resolved fluorescence studies of three-layered nanohybrids (C_60_Im→ZnP(alkyl)_4_ /SWCNT) gave further shortening of the fluorescence lifetimes (τ_F_) of the ^1^ZnP*(alkyl)_4_, giving the total k_CS_ values are (8–9) × 10^9^ s^−1^, from which the k_CS_ values from ^1^ZnP*(alkyl)_4_ to C_60_Im to form C_60_^∙−^Im→ZnP^∙+^(alkyl)_4_ are evaluated to be (2–3) × 10^9^ s^−1^.

### 4.2. Evidence for Photoinduced Charge Separation

**Figure 8:**
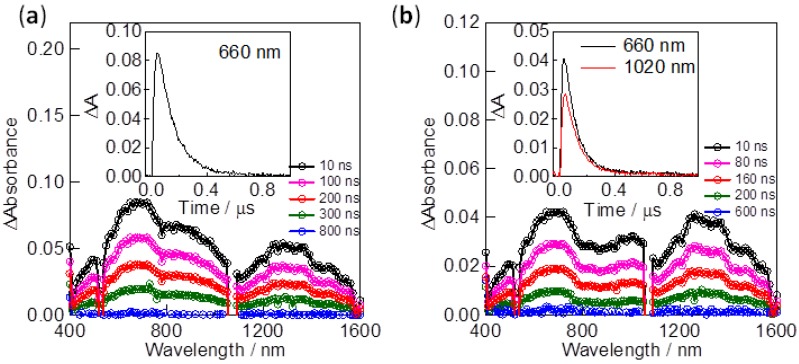
Nanosecond transient absorption spectra observed by 532 nm laser-lightpulse irradiation; (**a**) ZnP(alkyl)_4_/SWCNT(6,5)in Ar-saturated DMF and (**b**) C_60_Im→ZnP(alkyl)_4_/SWCNT(6,5)in Ar-saturated *o*-DCB: Inset, Absorption-time profiles(adopted from [48]).

Information for the CS products and kinetics of CR are obtained from the transient absorption spectral studies. Figure 8a shows the nanosecond transient absorption spectra of the ZnP(alkyl)_4_/ SWCNT(*n*,*m*) nanohybrids observed using a 532 nm laser light to excite selectively the ZnP(alkyl)_4_ moiety in Ar-saturated DMF. Transient absorption bands appear in the wide region from the Visible region to the NIR region. The absorption bands at 650 nm can be attributed to the ZnP^∙+^(alkyl)_4_ moiety. Since the NIR-band intensities decrease in almost the same rates with the visible ZnP^∙+^ bands, they can be thought as a pair of the RIP. Thus, the absorption bands at 900, 1,250 and 1,380 nm can be ascribed to the SWCNT(6,5)^∙−^.

In the case of ZnP(alkyl)_4_/SWCNT(7,6), the absorption bands of the ZnP^∙+^(alkyl)_4_ moiety similarly appear at 650 nm in addition to the broad NIR bands in 1,200–1,500 nm, whereas the 900 nm band is not found, suggesting that the broad 900-nm band in Figure 8a is one of the absorption bands of SWCNT(6,5)^∙−^. These assignments afford evidence for the occurrence of the CS process in the supramolecular nanohybrid to form ZnP^∙+^(alkyl)_4_/SWCNT^∙−^. The time profile of ZnP^∙+^(alkyl)_4_ at 650 nm is shown in the inset of Figure 8a. The rise of ZnP^∙+^(alkyl)_4_ is fast within the 6-ns laser light pulse, which corresponds to the fast CS rate within ca. 200 ps as estimated from the fluorescence lifetimes of ZnP(alkyl)_4_. The decay of the ZnP^∙+^(alkyl)_4_ mostly persists until about 300–400 ns; from the first-order fitting to the decay curve, the *k*_CR_ value is evaluated to be 6 × 10^6^ s^−1^. From the *k*_CR_ value, the lifetimes of ZnP^∙+^(alkyl)_4_/SWCNT^∙−^ (*τ*_RIP_) can be calculated to be 140 ns in DMF. Similar values are obtained from the decay-time profiles at the NIR bands. The *k*_C__S_/*k*_CR_ ratios, which are proportional to the charge stabilization, are 600~700 for ZnP(alkyl)_4_/SWCNT(*n*,*m*); these values are considerably large enough to prove the charge stabilization in DMF. 

Following is a comparison of kinetics of CS and CR with other kinds of ZnP/SWCNTs using SWCNT(6,5) and SWCNT(7,6). In the case of nanohybrids formed using ZnP covalently bonded with four pyrene moieties [(ZnP(Pyr)_4_], which attaches on SWCNT surfaces, *k*_CS_ = (3–4) × 10^9^ s^−1^, *k*_CR_ = (2–3) × 10^7^ s^−1^ (*τ*_RIP_ = 40–50 ns) and *k*_CS_/*k*_CR_ ≈ 150 [50]. For nanohybrids formed by ZnP coordinately bonded to an axial imidazole ligand tethered to a pyrene moiety (PyrIm→ZnP), in which the Pyr moiety attaches to the SWCNT surfaces (SWCNT/PyrIm→ZnP), *k*_CS_ = (5–6) × 10^9^ s^−1^, *k*_CR_ = (1–2) × 10^7^ s^−1^ (*τ*_RIP_ = 50–70 ns) and *k*_CS_/*k*_CR_ ≈ 500 [51]. For nanohybrids formed via ion-pairing, *viz*., ZnPs with charged groups at the macrocycle periphery attached to the opposite charged pyrene moiety on SWCNT surface, *k*_CS_ = (2–8) × 10^9^ s^−1^, *k*_CR_ = (0.5–0.8) × 10^7^ s^−1^ (*τ*_RIP_ = 50–70 ns) and *k*_CS_/*k*_CR_ ≈ 500 [52]. For ZnP with crown ethers complexed with ammonium ion-pyrene on SWCNT surface (SWCNT/Pyr-NH_3_^+^:CrownZnP), *k*_CS_ = (1–5) × 10^9^ s^−1^, *k*_CR_ = (0.9–1.0) × 10^7^ s^−1^ (*τ*_RIP_ = 90–110 ns) and *k*_CS_/*k*_CR_ ≈ 500 [53]. Clearly, these high *k*_CS_/*k*_CR_ ratios are favorable for further electron mediation and hole-shift, opening wider applications for solar photovoltaic cells and solar H_2_-evolution. 

On addition of C_60_Im in *o*-DCB, new band appears at 1,020 nm in addition to the Visible and NIR bands due to ZnP^∙+^(alkyl)_4_/SWCNT(6,5)^∙−^ as shown in Figure 8b. The 1,020-nm band is a diagnostic evidence of C_60_^∙−^Im, confirming the formation of C_60_^∙−^Im→ZnP^∙+^(alkyl)_4_/SWCNT(6,5) and C_60_^∙−^Im→ZnP(alkyl)_4_/SWCNT(6,5)^∙+^ via ^1^ZnP*(alkyl)_4_. The decay of C_60_^∙−^Im is almost the same as those of the visible band [ZnP^∙+^(alkyl)_4_] and the NIR bands [SWCNT(6,5)^∙+^].

Similar transient absorption spectral changes are observed for C_60_Im→ZnP(alkyl)_4_/SWCNT(7,6). The *k*_CR_ values evaluated from the 660 nm band and 1,020 nm bands become faster on coordination of C_60_Im, giving the larger *k*_CR_ values and shorter *τ*_RIP_ values; the faster CR process may occur between C_60_^∙−^Im→ZnP^∙+^(alkyl)_4_. A slight decrease in the *k*_CS_/*k*_CR_ ratios are induced on coordination of the C_60_Im due to structural and electronic reasons. It may be mentioned here that the decay curves of ZnP^∙+^(alkyl)_4_/SWCNT^∙− ^ and C_60_^∙−^Im→ZnP^∙+^(alkyl)_4_/SWCNT followed the trend expected for intra-molecular process and not intermolecular bimolecular process occurring on several μs time scales, suggesting that both ZnP(alkyl)_4_/SWCNT and C_60_Im→ZnP(alkyl)_4_/SWCNT are intact in solution during photochemical investigations [48].

### 4.3. Photoelectrochemical Studies

**Figure 9:**
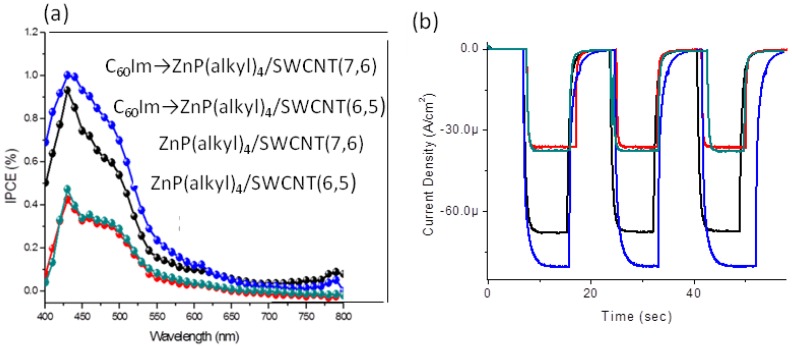
(**a**) IPCE spectra and (**b**) light-current switching curves for the photoelectrochemical solar cells of ZnP(alkyl)_4_/SWCNT and C_60_Im→ZnP(alkyl)_4_/SWCNT coated on SnO_2_-modified FTO electrode surface in acetonitrile containing 0.5 M LiI and 0.1 M I_2_ (I^–^/I_3_^−^) mediator. Red curve;ZnP(alkyl)_4_/SWCNT(6,5), black curve; C_60_Im→ZnP(alkyl)_4_/SWCNT(6,5), green curve; ZnP(alkyl)_4_/SWCNT(7,6), and blue curve; C_60_Im→ZnP(alkyl)_4_/SWCNT(7,6) (adopted from [47]).

The observed photo-induced CS processes in the investigated nano-hybrids prompted us to perform photoelectro-chemical studies to visualize their ability to convert the light energy into the electricity. For this, the ZnP(alkyl)_4_/SWCNT(*n*,*m*) and C_60_Im→ZnP(alkyl)_4_/SWCNT(*n*,*m*) nano-hybrids are drop-coated on SnO_2_-modified fluorine doped tin oxide (FTO) electrode surface, since this SnO_2_-modified FTO is known to yield high IPC with suitable porphyrins or phthalocyanines [54,55]. The photocurrent action spectra show IPCE values in the wide wavelength region covering up to 700 nm with maximum near 420 nm, which tracks the absorption spectra of the ZnP moiety (Figure 9a). The switching responses are shown in Figure 9b, in which quick response and light-stability are revealed. The higher IPCE values at 420 nm are obtained for C_60_Im→ZnP(alkyl)_4_/SWCNT(*n*,*m*) that for ZnP(alkyl)_4_/ SWCNT(*n*,*m*) by a factor of ca. 2, suggesting that C_60_Im contributes to the additional CS process via ^1^C_60_Im*. In C_60_Im→ZnP(alkyl)_4_/SWCNT(*n*,*m*), SWCNT(7,6) is slightly higher than SWCNT(6,5). 

In summary of this section, in the case of C_60_Im→ZnP/SWCNT(*n*,*m*) nanohybrids, further charge-separation from ^1^ZnP* to the C_60_ moiety takes place, in addition to from ^1^ZnP* to the SWCNT(*n*,*m*), giving larger *k*_C__S_ values than those of ZnP(alkyl)_4_/SWCNT(*n*,*m*). Photoelectrochemical studies of these nanohybrids modified on SnO_2_/FTO electrodes revealed photo-current generation. Higher IPCE values are obtained for C_60_Im→ZnP/SWCNT(*n*,*m*) than those of ZnP/SWCNT(*n*,*m*). 

## 5. Conclusions

In the present article, we have shown that supramolecular methods are simple and versatile approaches to construct donor-acceptor hybrids involving fullerenes to induce photoinduced events. Occurrence of photoinduced electron transfer is established by photophysical and photochemical methods such as fluorescence lifetime measurements and transient absorption spectral measurements. These phenomena are interpreted on the basis of the molecular orbital energies and electron-distributions calculated using DFT methods. The MO calculations of the supramolcular systems including the SWCNT(*n*,*m*) open a molecular paradigm of size-selected SWCNT from the material of SWCNT mixtures. Practical applications, especially in the area of photovoltaic cells and photocatalysis, including electron pooling and H_2_ production, are promising. Such applications are currently being explored in our laboratories. 
